# A quantitative study of letters to the editor by medical students in medical education journals

**DOI:** 10.1080/10872981.2021.1912879

**Published:** 2021-04-15

**Authors:** Anvarjon Mukhammadaminov, Sanketh Rampes, Yasmin Amy Divecha, Laura Leeves, David Hammond, Azeem Alam, Russell Hearn

**Affiliations:** a Faculty of Life Sciences & Medicine, King’s College London, London, UK; b Division of Anaesthetics, Pain Medicine and Intensive Care, Department of Surgery and Cancer, Faculty of Medicine, Imperial College London, London, UK; c School of Population Health & Environmental Sciences, Faculty of Life Sciences & Medicine, King’s College London, London, UK

**Keywords:** Medical education, letters to editor, medical student, uk medical schools, uk foundation programme

## Abstract

Letters to the Editor offer ways for readers to engage with authors’ publications. Letters are the shortest manuscript for medical students to publish and medical-education journals are best suited. The UK Foundation Programme rewards medical students achieving PubMed ID publications and we hypothesise that this is a main motivation for medical students to submit Letters to the Editor. A review of 15 medical-education journals with an impact factor was conducted to identify numbers and percentages of Letters to the Editor by medical students between July 2018 and June 2020. Affiliation of medical students was collected. Our results show over two years, 299 letters were published by medical students equating to 45.9% of total letters. There was a 60% overall increase in letters by medical students published in the first 12 months compared to second 12 months. During this period overall numbers of letters published increased by 27%. 86% of the letters published by medical students over the two-year period were from UK medical schools. Five schools accounted for 60.5% of these letters. The three medical schools with highest numbers of letters published were King’s College London, Imperial College London and University of Oxford for both 2018/19 and 2019/20. The increase in letters published overall with greater numbers published by students, may indicate greater awareness of Letters to the Editor as means of dissemination amongst medical students. UK medical schools published large numbers of letters, perhaps reflecting increasing importance to students of publications due to impacting on subsequent jobs. Results from our quantitative research revealing: large numbers of letters by medical students, increase in letters published from 2018/19 to 2019/20 and overrepresentation of UK medical students supports the hypothesis that medical students are publishing letters to achieve PubMed IDs. Further qualitative research is required to test our hypothesis.

## Introduction

A Letter to the Editor is a way for readers to engage authors on their publication and in the process add something important which could be interesting for others [1]. From reading medical education journals we noticed a considerably high number of letters by medical students. We hypothesised that medical students are publishing Letters to the Editor to obtain PubMed IDs. Publications which have a PubMed Identifier (ID) form part of application scoring for both the academic and standard UK Foundation Programme [2]. In the UK, after completing medical school, graduates enter the Foundation Programme, which is a national two-year, work–based training programme with the aim of bridging the gap between medical school and specialty/general practice training. It ensures that doctors develop the professional and clinical skills to prepare them for the next stage of training, whether that be core medical, core surgical, or specialty training, in line with General Medical Council (GMC) guidance [3]. At present, Foundation Programme applicants can obtain a maximum of 100 points; 50 points are from a national Situation judgment Test (SJT), and a further 50 points from an Education Performance Measure (EPM). For the EPM, 43 points are calculated from the student’s medical school ranking in deciles, and seven points are for educational achievements, of which two points are for publications [4]. To count as a publication, the medical student must be a named author on an article with a PubMed ID.

A Letter to the Editor is commonly the shortest type of manuscript published. Medical education journals are more approachable for medical students to submit Letters to the Editor, as their experience of medical school means they are often able to offer insights and perspectives into medical education. Amplifying student views and experiences has an important role in improving medical education and bridging the gap between educators and students [5]. It was decided to look at medical education journals published in English to quantify the percentage and number of Letters to the Editor by medical students. Medical student affiliations were analysed to identify any discernible patterns, both for UK and international medical schools.

## Aim

To analyse the number and origin of letters published by medical students in medical education journals.

## Hypothesis

UK medical students are publishing a large number of Letters to the Editor in medical education journals. One motivation behind publishing Letters to the Editor involves obtaining PubMed IDs for the UK Foundation Programme.

## Methods

To identify journals, four university library websites that listed medical education journals were searched: Imperial College London, McGill, Wayne State University and New York University [6–9]. Journals which did not have an impact factor were excluded as a method of quality control [10,11]. Journals which did not publish letters were also excluded. A list of predatory journals was compared to our list of journals[12]. Data were collected over the past two years from July 2018 to June 2020. For each journal, the following information was collected: number of issues published per year, whether the journal was indexed on PubMed, total number of letters and letters written by medical students. For letters, by UK medical students their affiliated medical school was noted and for letters by non-UK medical students their country of study was collected. To determine whether authors were medical students, affiliation was used and the letters were read to determine whether either the authors explicitly stated they were medical students or whether the letter was written from the perspective of a medical student. If unclear, authors were searched on ResearchGate and PubMed. If still unclear, authors were not counted as medical students. For the two journals with the highest number of letters by UK medical students, data were extracted for 5 years in total from July 2015 to June 2020.

## Results

### Search results

**Figure 1.**
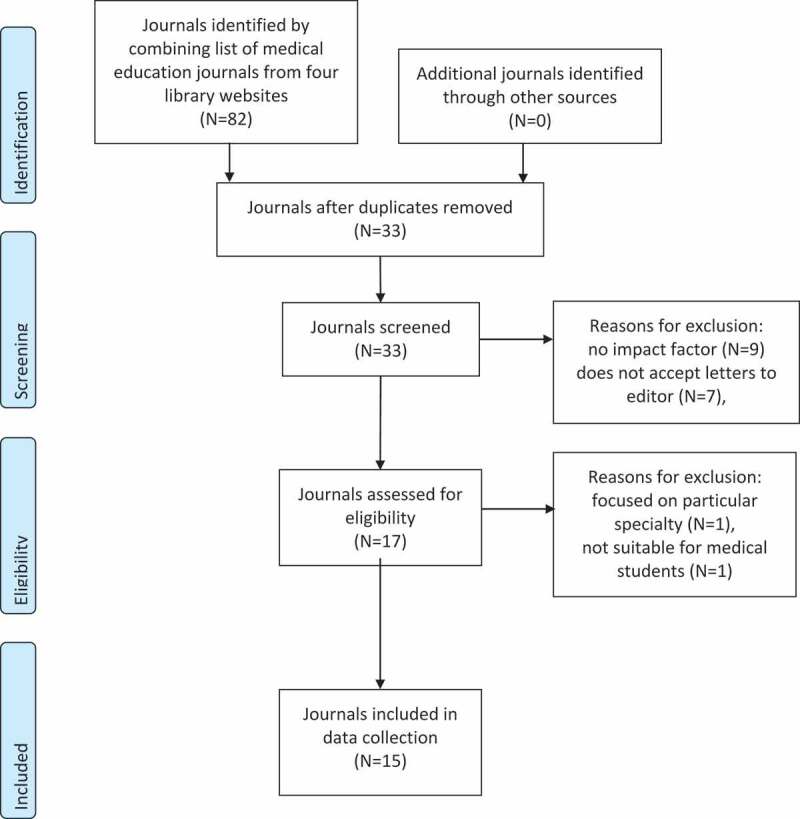
Flow chart for journal inclusion in systematic review

The initial list contained 33 medical education journals. Nine were excluded due to not having an impact factor. Seven were excluded as they did not accept Letters to the Editor. One was excluded as it was focused on a particular specialty and one was excluded due to being aimed at foundation year doctors. The final list included 15 journals, shown in Figure 1. Background characteristics of the journals are shown in Table 1. For all letters analysed we were able to determine whether authors were medical students. Letters to the Editor by non-medical students were written by doctors and medical educators.

**Table 1.** Baseline characteristics of medical education journals included in quantitative study

	Percentage of letters by medical students July 18 to June 20	Number of issues per year	Number of letters by medical students July 18 to June 20	Impact Factor	PubMed indexed?
The Clinical Teacher	83.3%	6	35	0.740 (2019)	Yes
Education for Primary Care	79.5%	6	31	0.590 (2019)	Yes
Medical Education Online	71.4%	1	30	1.970 (2019)	Yes
Medical Science Educator	70%	4	7	0.300 (2019)	Yes
Medical Teacher	69.8%	12	125	2.654 (2019)	Yes
Medical Education	48.8%	12	21	4.570 (2019)	Yes
Evaluation & the Health Professions	33.3%	4	1	1.578 (2019)	Yes
Simulation in Healthcare	25%	5	1	1.761 (2019)	Yes
Academic Medicine	18.2%	12	35	5.354 (2019)	Yes
Postgraduate Medical Journal	17%	12	8	1.911 (2019)	Yes
Anatomical Sciences Education	16.7%	6	1	3.759 (2019)	Yes
Journal of Graduate Medical Education	15.8%	7	3	0.649 (2019)	Yes
Education for Health	7.7%	3	1	0.74 (2018)	Yes
Advances in Physiology Education	0%	4	0	1.534 (2019)	Yes
Journal of Surgical Education	0%	6	0	2.220 (2019)	Yes

#### Quantity of letters by medical students and variation between journals

Over the two-year period, the total number of Letters to the Editor published by medical students in the 15 medical education journals was 299, equating to 45.9% of all Letters to the Editor published during the same period. The median number of letters published by medical students per journal was 7 (range 0 to 125) and median percentage of letters by medical students was 29% (range 0 to 83.3%).

Six journals had one or no letter by medical students published during the two-year period (Advances in Physiology Education, Anatomical Sciences Education, Journal of Surgical Education, Education for Health, Simulation in Healthcare and Evaluation & the Health Professions). One journal (Medical Teacher) published 125 letters by medical students which was 3.4 times higher than the number of letters published by the next highest journal, Academic Medicine, with 37 letters. Four journals had 70% or greater Letters to the Editor published by medical students (Medical Teacher, The Clinical Teacher, Education for Primary Care and Medical Education Online).

#### Change in letters published from 2018/19 to 2019/20

Total numbers of Letters to the Editor by medical students showed a 60% increase (115 to 184) from 2018/19 to 2019/20. This increase in letters by medical students was observed in all journals except two (The Clinical Teacher, Simulation in Healthcare). Over the same period the overall numbers of letters published increased by 28% (from 285 to 366).

#### Change in letters published from 2015 to 2020

For Medical Teacher the number of Letters to the Editor by medical students increased from 30 in 2015/16 to 73 in 2019/20. For The Clinical Teacher the number of Letters to the Editor by medical students increased from 9 in 2015/16 to 15 in 2019/20. The trend showing increasing Letters to the Editor published was more pronounced in Medical Teacher.

#### Seasonal variation in letters

**Figure 2.**
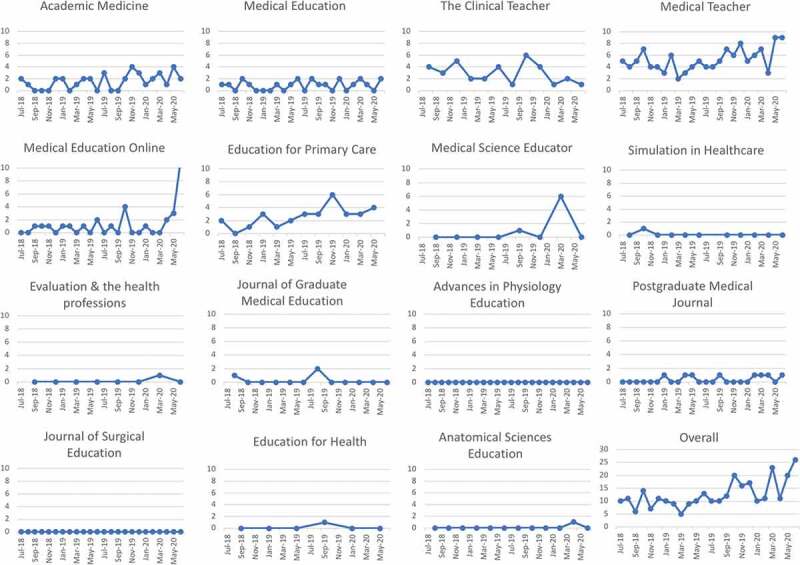
Line charts showing number of letters published by medical students

There was no obvious seasonal variation in letters published (Figure 2, supplementary Figure 1). Education for Primary Care demonstrated an increase in letters published in August 2018 and again in August 2019. The Clinical Teacher shows an increase in letters published around August 2019 (Figure 2).

#### Variance by medical school

**Figure 3.**
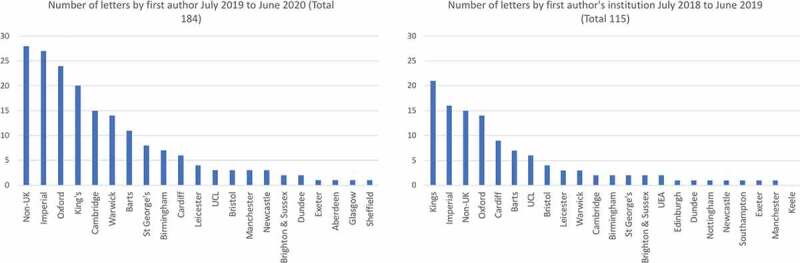
Bar charts showing number of letters published by medical schools

Over the two year period, some schools published consistently high numbers of papers; the three schools with the highest number of publications were: Imperial College London (43 letters, 13.7%), King’s College London (41 letters, 14.3%); and University of Oxford (38 letters, 12.7%) (Figure 3). Additional analysis adjusting for medical school size was conducted, using 2017 medical school intake figures from the Office for Students website [13]. The adjusted analysis for three medical schools with the highest number of letters for 2018/2019 remained unchanged, whereas for 2019/2020 University of Warwick was in the 3^rd^ place, above King’s College London.

There are 35 medical schools in the UK [14], yet from August 2018 to July 2020 five medical schools with the highest number of letters published 60.5% of total letters by medical students.

#### Affinity of certain medical schools for particular journals

For each journal, medical schools were ranked by number of letters published. Certain medical schools had the highest number of letters for the same journals for both years: King’s College London in Medical Education, University of Oxford in Medical Teacher, Imperial College London in Medical Education Online and University of Warwick/Imperial College London in Education for Primary Care (Table 2).

**Table 2.** Medical schools or countries ranked by total number of letters by medical students published in medical education journals

Journal Name	Medical school ranking by letters submitted 2018/19			Medical school ranking by letters submitted 2019/20		
	1st	2nd	3rd	1st	2nd	3rd
Academic Medicine	The USA	Imperial College London	N/A	The USA	Queen Mary University of London/ King’s College London/ Canada	Queen Mary University of London/ King’s College London/ Canada
Medical Education	King’s College London	University College London	Queen Mary University of London/ Leicester/ Bristol	King’s College London	St George’s University of London	Queen Mary University of London/ Imperial College London/ People’s Republic of China/ Brighton/Cambridge/UCL
Anatomical Sciences Education	N/A	N/A	N/A	People’s Republic of China/ St George’s University of London	People’s Republic of China/ St George’s University of London	N/A
Medical Teacher	University of Oxford	University of Cardiff	King’s College London	University of Oxford	University of Cambridge	King’s College London
Advances in Physiology Education	N/A	N/A	N/A	N/A	N/A	N/A
Journal of Surgical Education	N/A	N/A	N/A	N/A	N/A	N/A
Postgraduate Medical Journal	People’s Republic of China	The USA/University of Brighton	The USA/University of Brighton	St George’s University of London	Croatia/ People’s Republic of China	Croatia/ People’s Republic of China
Medical Education Online	Imperial College London	King’s College London	N/A	Imperial College London	King’s College London	University of Birmingham
Journal of Graduate Medical Education	The USA/ King’s College London	The USA/ King’s College London	N/A	The USA	N/A	N/A
The Clinical Teacher	King’s College London	University College London	Queen Mary University of London/ Imperial College London	Imperial College London	University of Oxford	University of Cambridge/ King’s College London
Education for Health	N/A	N/A	N/A	Canada	N/A	N/A
Education for Primary Care	University of Warwick/ Imperial College London/ King’s College London	N/A	N/A	University of Warwick/ Imperial College London	N/A	University of Oxford
Medical Science Educator	N/A	N/A	N/A	Imperial College London	King’s College London/ University of Warwick/ St George’s University of London	King’s College London/ University of Warwick/ St George’s University of London

#### Predominance of UK medical schools compared to non-UK schools

UK medical schools had, by far, the largest share of Letters to the Editor in the 15 international medical education journals studied. 86% of all letters published by medical students were by authors from UK medical schools in from June 2018 to July 2020. All except the following six journals had a majority of letters from UK medical students: Academic Medicine, Anatomical Sciences Education, Education for health, Journal of Graduate Medical Education, Postgraduate medical journal, Simulation in Healthcare. Apart from Academic Medicine however, these journals published a very low number of Letters to the Editor overall (four journals published three or fewer letters during the two-year period).

## Discussion

Our findings that large percentage of Letters to the Editor were published by medical students, and the majority of letters by medical students being from UK medical schools suggests that medical students are potentially writing Letters to the Editor as a means of obtaining a PubMed ID for Foundation Programme applications. One of our most interesting findings was that 85% of all Letters to the Editor published in medical education journals by medical students are written by UK medical students. Since we analysed medical education journals published in English, one might expect that medical students from all English-speaking countries would be equally likely to publish in these journals. This difference may reflect the differing value of publications in countries for applications. One clear exception was Academic Medicine which had a majority of letters from medical schools in USA, which may be explained by the fact that it is the official journal of the American Medical Association[15]. This results in a skewed, UK-centric perspective of students’ experience of medical education. Journals should identify ways to encourage participation from non-UK medical students.

The 60% increase in the number of Letters to the Editor by medical students published from 2018/19 to 2019/20, seems to suggest that more medical students are becoming aware of Letters to the Editor as a means of dissemination. Additionally, in the context of the COVID-19 pandemic, medical school placements were disrupted in many countries worldwide from February/March 2020 [16,17]. This may mean that medical students had additional time to develop their portfolios and submit articles, perhaps accounting for some of the increase seen in Letters to the Editor published. A longer period of data collection for all the journals will allow better determination of any possible impact of COVID-19.

Medical Education, Medical Education Online, Medical Teacher and Education for Primary Care were overrepresented by King’s College London, Imperial College London, University of Oxford and University of Warwick/Imperial College London, respectively. We hypothesised whether the editors publicised their journals to students at their university, however, we found no association between the editor or editorial board and the medical school producing the most letters. We believe that this finding may be explained by word of mouth; medical students are more likely to tell fellow students from their own university about submitting letters to a particular journal. Other factors which may account for certain medical schools disseminating a large amount of letters in medical education journals may include the amount of medical education teaching within the curriculum. For example, King’s College London has a module dedicated to medical education called ‘Doctor as a Teacher’ for third year medical students, which may boost students’ interest, awareness and engagement with medical education [18].

We expected to find seasonal variation in letters published by medical students, however we did not find any. This may be partially explained by the variance in delay between date of submission and date of publication. A limitation in our research is that we were only able to study letters published, we hypothesise that if we had data on submissions of letters this may follow a seasonal pattern with an increase in letters submitted around time of foundation application deadline, usually beginning of November[4]. We emailed all journals to enquire about submissions, only Education for Primary Care provided that information which showed a large increase in submissions coinciding with COVID-19.

A limitation of our study was that we restricted our review to medical education journals, and therefore are unable to say whether the same trend of medical student letters is seen in other types of journals. In addition, the data was only collected from journals published in English language which may have excluded letters from non-English speaking medical students. Another significant limitation was the short period of data collection. Ideally, we would have liked to collect data for a longer period of time, to allow identification of trends over time, however, we were limited by feasibility. Therefore, two journals, Medical Teacher and The Clinical Teacher, with the highest number of Letters to the Editor by UK medical students were chosen and data was collected for an additional three years equating to a total period of five years. The data showed an overall increase in Letters to the Editor over the five year period, suggesting that the trend of increasing numbers of letters by medical students predates our period of data collection. We excluded journals without an impact factor as a form of quality control of journals [10,11]. Among those journals, there were some which were PubMed indexed, which means that some may have also had a large number of letters published by medical students.

On the 30 November 2020, the Medical and Dentral Recruitment and Selection (MDRS) Programme Board and the UK Foundation Programme Office (UKFPO) published a decision to remove Educational Achievement (EA) score from the application scoring criteria from the 2023 allocation process. Therefore publications will no longer count for points towards the application from 2023. There has been widespread criticism of this decision, with calls for the UKFPO to reconsider [19]. Should this decision go ahead as planned, this will serve as an ideal opportunity to further test our hypothesis. A study in the form of an interrupted time series study can be designed before and after the 2023 application, to observe whether there will be a fall in Letters to the Editor published by medical students after 2023, as would be predicted by our hypothesis.

## Conclusion

Letters to the Editor are a valuable way for readers to engage with publications and contribute to the discussion of the topic. Publications with PubMed IDs count toward application for the Foundation Programme. Our quantitative study reveals a high number of Letters to the Editor by medical students, a rise in letters published by medical students from 2018/19 to 2019/20 and the overrepresentation of UK medical students. Although Letters to the Editor are used by readers to engage with authors and contribute new ideas, collectively the results from our quantitative study support our hypothesis that medical students are submitting Letters to the Editor to obtain PubMed IDs. Regardless of medical students’ intention for submitting Letters to the Editor, the process itself will promote engagement of medical students in medical education as well as helping them improve their academic skills, such as critical appraisal of articles. The nature of our quantitative research only allows us to identify trends in number of letters published. However, medical students’ intentions for submitting letters remain unclear. Future qualitative research in the form of anonymised questionnaires are required to test our hypothesis. We hope that more medical students from non-UK medical schools engage with the literature and publish letters to offer their insights of medical education from a non-UK perspective which will be beneficial for the global medical education community. Journals should seek methods to encourage engagement from non-UK medical students.

## Supplementary Material

Supplemental Material

Supplemental Material
