# Supplementary figures and images for: A quantitative study of letters to the editor by medical students in medical education journals
Source: Med Educ Online. 2021 Apr 15;26(1):1912879. doi: 10.1080/10872981.2021.1912879 (PMC8057084; doi:10.1080/10872981.2021.1912879)

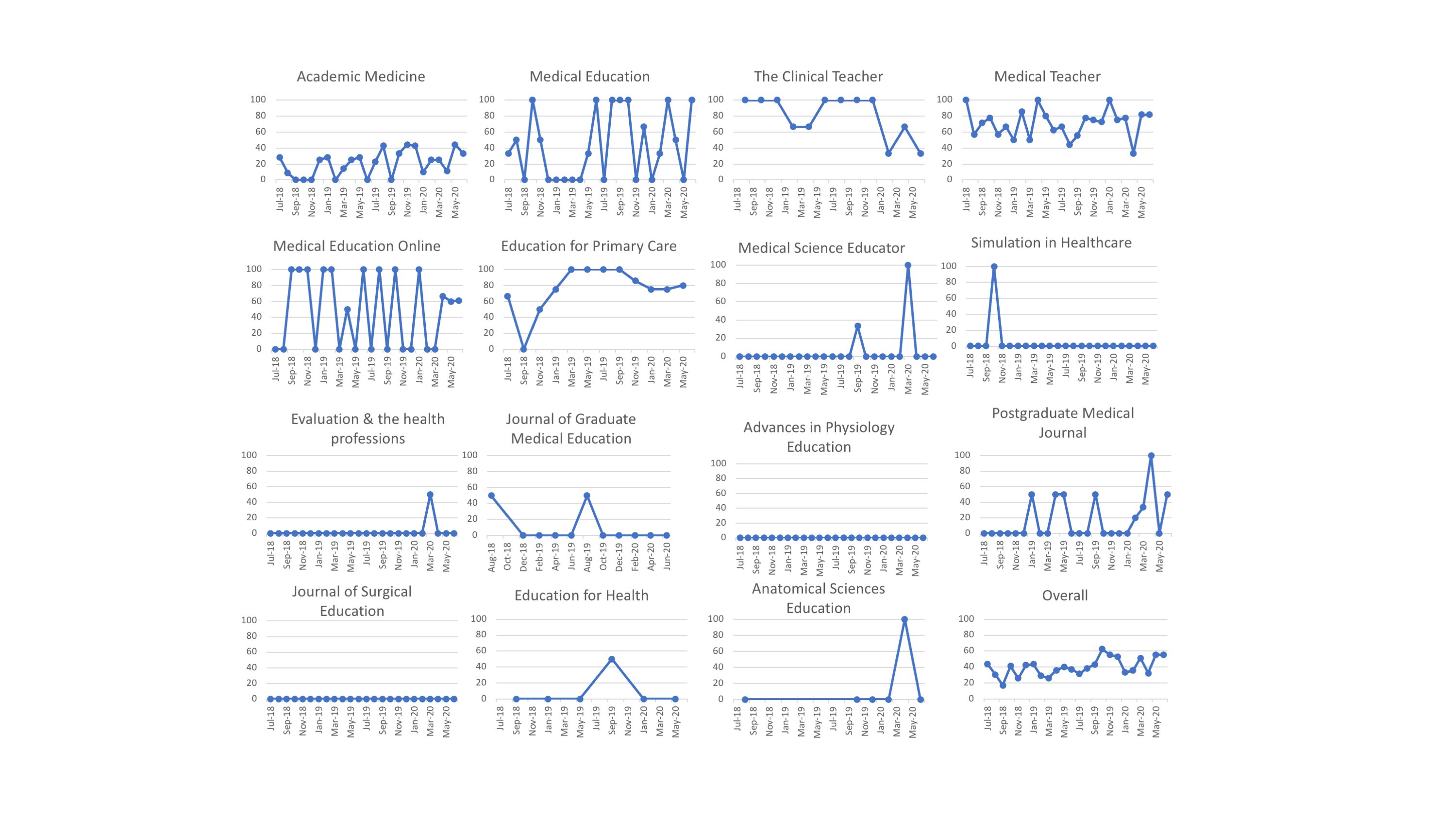

Supplement: Supplemental Material [file ZMEO_A_1912879_SM4140.tiff]
